# Information usefulness of public disclosure in Taiwan: Does it vary across specific diseases/conditions and contexts?

**DOI:** 10.1371/journal.pone.0310340

**Published:** 2025-03-28

**Authors:** Tsung-Tai Chen, Kai-Ren Chen, Ming-Hsin Phoebe Chiu, Chih-Kuang Liu, Wei-Chih Su, Vinchi Wang

**Affiliations:** 1 Department of Public Health, Fu Jen Catholic University, New Taipei, Taiwan, R.O.C; 2 Graduate Institute of Library and Information Studies, National Taiwan Normal University, Taipei, Taiwan, R.O.C; 3 Department of Urology, Camillian Saint Mary’s Hospital Luodong, Yilan, Taiwan, R.O.C; 4 Graduate Institute of Business Administration and College of Medicine, Fu-Jen Catholic University, New Taipei, Taiwan, R.O.C; 5 Department of Gastroenterology, Taipei Tzu Chi Hospital, Buddist Tzu Chi Medial Foundation, New Taipei City, Taiwan, R.O.C; 6 School of Medicine, College of Medicine, Fu Jen Catholic University, New Taipei, Taiwan, R.O.C; 7 Department of Neurology, Cardinal Tien Hospital, New Taipei, Taiwan, R.O.C; Almoosa College of Health Sciences, SAUDI ARABIA

## Abstract

**Objectives:**

This study discusses issues regarding tailored information for report cards, including what kinds of information patients with different diseases need and how the necessary information changes for these patients given alterations to a specific context. This study aimed to determine whether there is consistent, essential quality information across different diseases and in diverse contexts. The priority of needs related to interpersonal and technical quality information for different diseases is also discussed.

**Methods:**

Fifty-five patients from 5 hospitals in Taiwan were interviewed or invited to participate in a focus group. Patients were diagnosed with five different diseases or conditions: stroke, dialysis, AMI, diabetes, and knee problems. We conducted in-depth interviews to identify the most requested types of information for every disease or condition in general and in different contexts (e.g., relocation). We applied the Kano model to verify the relative priority of the information that emerged from the interviews for each disease.

**Results:**

The 3 most requested types of information among patients with various diseases or conditions in the general context were medical professionalism, physician communication skills, and accessibility. Only a few types of information were valued by patients with specific diseases. In addition, patients focused on specific and mutually relevant information in certain contexts (e.g., in the context of conflict with physicians, patients considered communication skills most important). This information was similar to the 3 most common types of information in the general context regardless of the disease, with the exception of stroke. Finally, technical quality information was treated as basic or necessary information. However, most important information was treated as expected information regardless of the disease.

**Conclusions:**

There is somewhat consistent essential quality information across different diseases and diverse contexts. According to the results of the Kano model, the report card should disclose interpersonal and technical quality simultaneously.

## 1 Introduction

Quality report cards serve as a quality improvement tool by encouraging patients to make informed decisions based on the information provided in the report cards, which can help patients choose healthcare facilities that offer high-quality services [1, 2]. Some studies have shown that quality report cards can lead to overall improvements in healthcare quality [3]. However, several systematic studies have demonstrated the ineffectiveness of official report cards [4, 5], as have patient-reported outcome measures [6]. Tailored quality information for report cards has been proposed as an important strategy to enhance their effectiveness [7, 8]. Faber et al. (2009) proposed a sequential improvement approach for report cards and suggested that for a report card to be successful, patients must undergo four stages: awareness, knowledge generation, attitude formation, and spontaneous use of the report card [7]. Subsequently, Sandmeyer and Fraser provided a similar framework for successful report card design [8]. Both the concepts proposed by Faber et al. and those proposed by Sandmeyer et al. share commonalities, such as identifying information content preferred by patients and providing comprehensible information or knowledge based on patient preferences. Studies related to tailored quality information have focused on comparing the needs of patient groups that differ in terms of gender [9], socioeconomic status [10, 11], behavior [11], ethnicity [12], stakeholders [13], setting (i.e., primary care) [14], country [15], context (e.g., increased comorbidity) [16–18], and diseases/conditions/surgeries [1, 11, 12, 16, 19–25]. Many studies have assessed patient demand under only a single condition, disease or surgery. Few researchers have compared the needs of patients with different diseases, and these studies have reported inconsistent results. One study revealed that patients with different conditions or diseases have different needs, such as effectiveness and safety for knee arthrosis, continuity of care and a relationship with therapists for chronic depression, and expertise for Alzheimer’s disease [22]. Other studies have revealed that patients’ required information is potentially invariant across different disease categories [23, 25, 26]. However, these studies did not discuss patients’ needs in terms of finding appropriate hospitals or physicians through report cards or physician rating websites, with the exception of one article [26]. Furthermore, very few studies have compared the needs of patients with different diseases in the context of finding appropriate hospitals or physicians in different contexts, such as changes in residence or different levels of disease severity [16]. Finally, in addition to exploring the consistency of report card content across different diseases [27], it is essential in practice to determine the priority sequence (the type of quality attribute) for implementing this content.

According to the Kano model, we aimed to determine the priority level of patients’ needs across different diseases. For example, a key consideration is whether patients view information—whether technical or interpersonal quality—as their top priority (basic quality) that a physician should possess. A detailed explanation of the Kano model can be found in the S1 Appendix. Hence, the objectives of this study are to (1) determine the consistency of essential quality information across different diseases and contexts, and (2) assess the priority of interpersonal and technical quality information for patients with varying conditions.

## 2 Materials and methods

### 2.1 Research participants

Fifty-five patients with 1 of 5 different diseases or health conditions were recruited from 5 hospitals between August and October 2018 and were referred by physicians from different specialties. The patients were selected through a purposive approach. The 5 diseases or health conditions included diabetes, dialysis, acute myocardial infarction (AMI), stroke, and knee problems (underwent total knee replacement [TKR]). We chose these diseases and medical issues because they were all on the list of official public report cards [28].

### 2.2 Researcher qualifications

Three professional researchers (the authors), who are associated professors with PhDs and teach at universities, individually applied thematic analysis and qualitative methods to transcribe and code the text, by quality information, onto the list (themes) mentioned below. Two of the researchers have more than 10 years of experience with qualitative studies in the fields of online reviews and behavioral science. One researcher has extensive experience with medical quality, especially regarding report card designs and evaluations.

### 2.3 Data collection—principles and steps

The research paradigm was constructivist. The qualitative studies moved through 2 stages because we applied the constant comparative method to elicit important quality information [29] as a base (first stage, in-depth interview). Then, through continuous stakeholder engagement (second stage, focus group) as suggested by dosReis et al. (2016), we verified the quality information generated by the researcher from the first stage [30]. The qualitative results from the interviews, which were based on coding by experts, may not be valid. The challenges that experts may face in understanding patients’ perspectives are understanding or empathizing with the experiences, feelings, and perspectives of patients. Through a sensitivity check for the needs proposed by patients in the in-depth interviews (continuous stakeholder engagement), it is possible to understand how experts can put themselves in the position of patients to gain insight into their challenges, needs, and concerns.

The basic steps are as follows: during the first stage, we conducted in-depth interviews that lasted approximately one hour to elicit information from patients with certain diseases or conditions in the general context and information in three different contexts (see below). In the second stage, we completed two tasks via the focus group meetings. The first was to ask participants to map the statements that the patients made in the interviews onto certain themes in order to calculate the sensitivity (see below). The second task was to apply the Kano model (see below) to verify the priority level of the information that emerged from the interviews for every disease or condition. The details regarding these two stages are provided below.

Before the interview procedure, we conducted a pretest with 5 patients to gauge the appropriateness of the procedure and interview process. The interview included five detailed steps: 1) A detailed explanation of our final goals (i.e., building reliable physician rating websites with rich content in the future) and the reasons for doing the research. 2) We presented the results of official report cards as examples to introduce the participants to quality differences. This step was vital because research has demonstrated that most patients do not believe that there are different levels of care quality across facilities and physicians [1, 31], and approximately 35% of adults perceive significant differences between hospitals, according to a nationwide survey [32]. 3) We provided pictures from NHS Choices and explained their purpose so that the patients understood what a public disclosure website was, and they were informed that any type of quality information could be disclosed since the patients lacked experience using official public report cards or physician rating websites in Taiwan [33]. 4) Regarding the composition of quality information, we not only applied the qualitative approach to retrieving possible information but also referred to existing disclosed quality information and literature reviews for eliciting as many themes of quality information as possible. The disclosed quality information was obtained from the Taiwanese official report card [28], National Quality Forum (NQF) [34], Hospital/Physician Compare [35], and NHS Choices [36]. We rewrote these technical measures retrieved from these sites in a clear and understandable way, reflecting the potential impacts on patient outcomes. The information from the literature review was derived from the Health Information Wants Questionnaire (HIWQ) [37, 38]. 5) Finally, we assumed 3 kinds of contexts and asked the same questions as above to identify the information within three contexts, including relocations or job changes, increased comorbidities and severities, and conflicts with physicians [16]. Before asking the questions in step 5, we first ensured that the patients were likely to change physicians in any context. This was achieved by asking whether patients were able to change physicians. If they said this was possible, we proceeded with step 5.

The second-stage focus groups included 5 to 6 patients with different diseases or primary caregivers who were not the same persons who had participated in the in-depth interviews discussed above. In this stage, we allowed the primary caregiver at home to attend the meeting for the patient because we wanted to ensure that everyone in the meeting, especially elderly individuals, comprehensively understood the exact meaning of every theme derived from the in-depth interviews. We omitted patients or caregivers related to TKR because we did not gather sufficient participants. The details regarding these focus groups are provided below. We asked participants with 4 different diseases to map the statements that the previous patients made in their interviews onto certain themes (i.e., medical professionalism) in order to calculate sensitivity. Each theme was presented to the patients on a card that provided a detailed description of its actual definition, given in the Results section. We randomly selected one complex statement from the in-depth interviews for the 3 most important kinds of information for every disease and then checked them against the themes proposed by the focus group participants.

We applied the Kano model to verify the priority level of the information derived from the in-depth interviews. The retrieved information included technical and interpersonal quality information. However, most patients may not pay much attention to these clinical process measures [39] because it is difficult to understand their meaning [40]. Therefore, the information elicited from patients in the interviews may not be of technical quality. If there was no technical quality information retrieved among the important themes in the in-depth interview, we added two common technical quality information points: whether the physicians actually performed the necessary examinations or prescribed the appropriate medications.

### 2.4 Data analysis

Data saturation was achieved during analysis of the 6th interview transcriptions of every disease and condition, and 1 or 2 additional interviews were conducted to ensure that no additional themes emerged. The interviews were recorded as transcriptions in Microsoft Excel. If there were inconsistencies in the coding, the researchers discussed the differences. After discussion, the coding was abandoned or reorganized. Regardless of any decision, the researchers sought to achieve 100% consistency. Finally, the research team rereviewed the adjusted coding according to the recorded files to avoid omitting any information. If there were any omissions, a new code was formed. After the interviews, we listed the 3 most important (most frequently mentioned) themes for every disease or problem condition.

This research was approved by the ethics committee of the institutional review board of New Taipei City Hospital (IRB Number 107002-E). All participants provided informed, written consent, while the data security and anonymization or deidentification methods met the requirements of the institutional review board.

## 3 Results

As shown in Table 1, 55 patients (or their caregivers) participated, including 13 with diabetes, 12 with AMI, 12 undergoing dialysis, 12 with stroke (poststroke patients) and 6 with TKR. Among the patients with diabetes or their caregivers, the average age was 62, and 60% were female (8/13); 7 patients participated in the in-depth interviews. Among the patients with AMI or their primary caregivers, the average age was 55 years, and 80% were male (10/12); 6 patients participated in the in-depth interviews. Among the patients undergoing dialysis or who were their primary caregivers, the average age was 53, and 70% were male (8/12); 7 patients participated in the in-depth interviews. Among the patients with stroke or their primary caregivers, the average age was 60, and 70% were male (8/12); 7 patients participated in the in-depth interviews. Among the patients who underwent TKR, the average age was 68 years, and 65% were male (4/6).

Below, we discuss the following: the three most important pieces of information for patients with any disease or condition in the general context; specific information for patients with a specific disease or condition in the general context; the most important information for patients with any disease or condition in a specific context; mapping the statements onto certain themes (sensitivity check); and the Kano model to verify the priority of the information.

**Table 1:** Age and gender of patients who participated in the in-depth interviews and focus groups.

In-depth interview				Focus group						
ID	Disease or procedure	Gender	Age	ID	Disease	Gender				Age
D01	Diabetes	Male	30	D08	Diabetes		Female			58
D02	Diabetes	Male	46	D09	Diabetes		Female			66
D03	Diabetes	Female	59	D10	Diabetes		Female			71
D04	Diabetes	Male	52	D11	Diabetes		Female			67
D05	Diabetes	Female	54	D12	Diabetes		Male			67
D06	Diabetes	Male	44	D13	Diabetes		Female			77
D07	Diabetes	Female	35							
M01	AMI	Male	42	M07	AMI		Male			55
M02	AMI	Male	52	M08	AMI		Male			57
M03	AMI	Male	60	M09	AMI		Male			55
M04	AMI	Male	75	M10^a^	AMI		Female			53
M05	AMI	Male	51	M11	AMI		Male			69
M06	AMI	Male	54	M12^a^	AMI		Female			39
H01	Dialysis	Female	47	H08	Dialysis		Male			65
H02	Dialysis	Male	65	H09	Dialysis		Male			55
H03	Dialysis	Male	50	H10^a^	Dialysis		Female			59
H04	Dialysis	Female	53	H11	Dialysis		Male			47
H05	Dialysis	Male	56	H12	Dialysis		Female			60
H06	Dialysis	Male	39							
H07	Dialysis	Male	40							
S01	Stroke	Female	51	S08	Stroke		Female			52
S02	Stroke	Male	52	S09	Stroke		Male			42
S03	Stroke	Male	72	S10^a^	Stroke		Male			79
S04	Stroke	Male	61	S11^a^	Stroke		Male			68
S05	Stroke	Female	74	S12	Stroke		Male			61
S06	Stroke	Female	68							
S07	Stroke	Male	43							
T01	TKR	Male	64							
T02	TKR	Male	73							
T03	TKR	Female	79							
T04	TKR	Male	67							
T05	TKR	Male	60							
T06	TKR	Female	62							

Note.

^a^: Primary caregiver at home; AMI: acute myocardial infarction; TKR: total knee replacement; no TKR focus group was held.

### 3.1 Three most important pieces of information for patients with any disease or condition in the general context

The 3 most valued types of general information for every disease or condition (general context) were medical professionalism, physician communication skills, and accessibility. Examples from the interview transcripts are also listed below (H represents patients on dialysis, S represents patients with stroke, D represents patients with diabetes, M represents patients with AMI, and T represents patients who have undergone TKR).

## 3.1.1 Medical professionalism

Patients with different kinds of diseases identified "medical professionalism" as one of the most important themes. In the general context, more than 75% (24/33) of patients with different diseases felt it to be important. Patients felt that the definition of "medical professionalism" varied with the disease but generally referred to having 4 subthemes, including good medical skills (with cross-specialist knowledge) for effective treatment, integrative care/multidisciplinary team care, treatment efficiency, and physician surgery volume.

*3*.*1*.*1*.*1 Effective treatment*. Effective treatment is about being able to effectively solve the patient’s problems (improved health status or success rate of treatment) and prevent subsequent adverse events. For example, in the case of stroke, patients felt that improving their condition was important, as was preventing their disease from becoming severe, which refers to the need for medical personnel to suppress thrombosis at its first appearance.

H06: 「*It is about the effectiveness of the medical treatment*.」
S07: 「*Professionalism means faster recovery from illness or avoiding subsequent adverse events*.」
T04: 「*There are only two months left*, *and I know I have less chance to completely recover because I still feel swelling numbness in my legs*.」

*3*.*1*.*1*.*2 Integrative care/multidisciplinary team care*. Regarding integrative care/multidisciplinary team care, patients with diabetes believe that endocrinologists need to have the ability to manage diabetes throughout their lifespan. In addition, the doctor’s specialty may need to include sports therapy. Patients undergoing dialysis felt that it was important to have cross-specialty knowledge (e.g., nephrology and cardiology).

H03:「*When I was in the intensive care unit*, *the cardiologist told me not to drink water*, *but the nephrologist told me to drink water*.」
D04: 「*When I started slowly increasing to a large amount of exercise*, *I went to another hospital to find a doctor specializing in sports therapy*.」

*3*.*1*.*1*.*3 Treatment efficiency*. In the case of AMI, patients defined the level of medical professionalism based on the ability to provide timely treatment. For example, nurses on medical teams should act quickly to rescue patients with AMI.

M02: 「*My wife just saw the nurses running and pushing me in a wheelchair*. *My wife was moved to tears*.」

*3*.*1*.*1*.*4 Physician surgery volume*. In the case of TKR, patients considered a surgeon who had experience treating many patients to be specialized in conducting TKR because they may consider a surgeon to have a good reputation if he or she had many patients to see.

T02: 「*The greater the patient queue length is*, *the better the reputation the surgeon has; however*, *this does not necessarily mean he is a specialized surgeon*.」

### 3.1.2 Physician communication skills

More than 70% (22/33) of patients with different diseases felt that communication skills were important. Patients felt that the definition of "physician communication skills” generally referred to having 2 communication subthemes, including content and attitude. The important communication content included information on drug use, risks and uncertainties, ease of understanding, and communication attitudes including sufficient consultation time and careful listening.

*3*.*1*.*2*.*1 Communication content*: *Drug use*. Regarding drug use, for example, in the case of stroke, one patient suggested that the doctor should use simple words to explain the patient’s condition or its severity, especially to explain the anticoagulant medication, the reason why this drug was prescribed and its side effects (e.g., bleeding).

S01: 「*The doctor did not explain the limitations of anti-thrombotic medications for menstruating women*, *and she almost developed metrorrhagia*.」
S03: 「*If the doctor does not explain clearly*, *the family members may panic of seeing red urine*, *which perhaps indicates bleeding*.」
D03: 「*Because patients usually have multiple diseases and take different drugs*, *they sometimes do not know what the right ways are to take different pills*.」

*3*.*1*.*2*.*2 Communication content*: *Risks and uncertainties*. Regarding risks and uncertainties, there was uncertainty about the treatment (a gray area). It is possible that the physician could not confidently assure patients by answering their questions. However, from the aspect of patients, they expect doctors to answer their questions with certainties.

H03: 「*I asked the attending physician about how much water I can drink in a day*. *The doctor never answered because he said that he is uncertain*.*」*

*3*.*1*.*2*.*3 Communication content*: *Easy to understand*. For example, because most dialysis patients are elderly people, they want their doctor to talk to them in a way they can understand, and they want to be understood in turn. In this way, two-way interaction is achieved.

H07: 「*If the doctor and I can’t understand the words spoken by each other…*, *I will be angry when the doctor keeps asking questions*.」

*3*.*1*.*2*.*4 Communication attitude*: *Sufficient consultation time*. Regarding sufficient consulting time, for example, the dialysis patients thought that because physicians had many patients, they did not interact with their patients well.

H02: 「*The doctor may not have time to tell you directly*. *You need to have extra time to ask the doctor questions*, *but he doesn’t have that available time*.」

*3*.*1*.*2*.*5 Communication attitude*: *Careful listening*. Because dialysis is a lifelong treatment, when young people are put on dialysis, they have many questions to ask. Therefore, such patients care whether their doctor is attentive to their concerns.

H06: 「*Because I had to have dialysis at a young age*, *I want doctors to listen to my thoughts*. *I sometimes have some worries*.」

## 3.1.3 Accessibility (Facility distance)

Forty-five percent (15/33) of patients listed accessibility as an important attribute. Patients who have had a stroke or AMI may choose to go to the closest hospital if they have a recurrence, and they may also choose to rehabilitate at a hospital near home. Because dialysis patients must go to the hospital three times a week, they wish to visit a hospital near home.

### 3.2 Specific information for patients with a specific disease or condition in the general context

Below will discuss health promotion for patients with diabetes or stroke and facility fame and reputation and emotional support for patients undergoing TKR.

## 3.2.1 Health promotion/health education by health educators or physicians for patients with diabetes and stroke

Compared with other diseases, diabetes treatment often involves not only physicians but also health educators, dietitians and social workers. Patients with diabetes considered health education to be the most important quality information attribute, preferring it to be provided by teams.

D03: 「*Every day you need to take care of your health through diet*, *medicine and*

*exercise*. *I think this is basic knowledge that is needed and important*.」

D07: 「*The nurses or doctors in this hospital will encourage patients to control their*

*blood sugar level in a successful way*.」

D07: 「*A good doctor can help the patient learn and understand how to manage diabetes for a lifetime*.」

In contrast to those with diabetes, patients who have had strokes expressed that health promotion and health education were important but should be conducted by physicians. Patients with stroke and their family members expressed anxiety about a subsequent stroke. Therefore, the patients or family members suggested that before patients leave the hospital, the physician should provide them with relevant knowledge in the inpatient setting, including information on follow-up treatment and self-management for stroke treatment (rehabilitation, medication, etc.). Otherwise, the education materials could be posted on a website to allow patients and family members to access relevant electronic resources. One patient who had a stroke stated that his evaluation of a hospital was based to a great extent on the amount of website education resources.

S02: 「*When I was going to leave the hospital*.*…*. *It would be useful to tell me the rehabilitation that I need to do at home*.」
S03: 「*We are laymen*. *The doctor is a professional*. *He should tell the patient in detail the patient’s current health and treatment of the disease*.」
S04: 「*The doctor just said that the cerebral blood vessels were getting smaller*, *but he did not say anything else*! *What is the follow-up treatment and rehabilitation*? *Should I continue to take medicine*?」
S03: 「*Health education materials are the relevant disease information I need…*. *If the hospital does not have health education materials*, *the hospital sucks*.」

## 3.2.2 Facility fame, reputation and emotional support for patients undergoing TKR

Patients who underwent TKR were more willing to search for surgeons specializing in orthopedics and needed emotional support to make social and psychological adjustments.

T01: 「*We often searched Google to find doctors with an orthopedics specialty first and then checked their expertise in orthopedics*.」

T01: 「*It is important to provide emotional comfort for patients because after surgery*, *they are unable to move or need to stay in bed for several days*」

### 3.3 Most important information for patients with any disease or condition in a specific context

For the specific contexts, we listed only the most important attributes that at least two patients mentioned because there were many themes that were mentioned by only one patient. As shown in Tables 2 and 3, when moving to new homes or changing jobs, patients with different diseases or conditions, except stroke, mentioned that accessibility was most important, while in the context of increased comorbidity or severity, they considered medical professionalism to be the most important, and in the context of conflict with physicians, most of them considered communication skills the most important and the relevant theme of physician attitude the second most important. Furthermore, the themes of facility/physician reputation and fame were valued in every specific context by patients with chronic diseases or conditions, such as diabetes and dialysis.

**Table 2:** Attributes considered important by patients with different diseases or conditions in the general context or specific contexts: Change in residence or job.

	General context					Specific context: Change in residence or job				
	Stroke	Dialysis	AMI	Diabetes	TKR	Stroke	Dialysis	AMI	Diabetes	TKR
Medical professionalism	1 (6/7)	1 (5/7)	1 (5/6)	2 (4/7)	2 (4/6)	1 (4/7)				
Communication skills	2 (5/7)	1 (5/7)	3 (3/6)	2 (4/7)	1 (5/6)					
Facility distance	2 (5/7)	1 (5/7)	1 (5/6)			2 (3/7)	1 (4/7)	1 (3/6)	1 (4/7)	1 (2/6)
Health education (by physician)	1 (6/7)			2 (4/7)						
Health education (by medical staff)				1 (5/7)						
Facility/physician reputation					2 (4/6)		2 (3/7)		2 (2/7)	
Emotional support and social and psychological adaptation					2 (4/6)					

Note. Only the top 3 quality information needs are listed for the general context; we listed only the top important attributes that at least two patients mentioned in the specific context; AMI: acute myocardial infarction; TKR: total knee replacement

**Table 3:** Attributes considered important by patients with different diseases or conditions in specific contexts: Increased disease complexity or conflict with physicians.

	Specific context: Increased severity or comorbidity						Specific context: Conflict with physicians				
		Stroke	Dialysis	AMI	Diabetes	TKR	Stroke	Dialysis	AMI	Diabetes	TKR
Medical professionalism		1 (7/7)	1 (4/7)	1 (2/6)	1 (4/7)	1 (3/6)	2 (3/7)					
Communication skills							1 (5/7)	1 (4/7)	1 (2/6)	1 (4/7)		
Facility distance												
Health education (by physician)												
Integrative care or multidisciplinary team care			2 (3/7)									
Physician attitude								2 (2/7)	1 (2/6)	2 (2/7)	1 (2/6)	
Facility/physician reputation					3 (2/7)			2 (2/7)				
Physician trust					2 (3/7)							

Note. We listed only the most important attributes that at least two patients mentioned in the specific context; AMI: acute myocardial infarction; TKR: total knee replacement

### 3.4 Mapping the statements onto certain themes (sensitivity check)

In Table 4, the sensitivity check based on the focus group data for diabetes, AMI, dialysis, and stroke yielded values of 0.78, 0.78, 0.67, and 0.67, respectively.

**Table 4:** The sensitivity check in the focus group for the needs proposed by patients in the in-depth interviews.

	Stroke	Dialysis	AMI	Diabetes
Medical professionalism	3/6	4/5	4/6	4/6
Communication skills	5/6	3/5	6/6	6/6
Health education ^a^	4/6			4/6
Emotional support and social and psychological adaptation		3/5		
Facility/physician reputation			4/6	
Sensitivity	0.67 (12/18)	0.67 (10/15)	0.78 (14/18)	0.78 (14/18)

Note: ^a^ Health education provided by physicians or medical staff

### 3.5 Kano model to verify the priority of the information

In Table 5, regarding the theme priorities of patients with different diseases or their primary caregivers, using the Kano model, we found that most of the general themes in Table 2 were expected themes rather than basic (necessary) themes. The basic (necessary) themes observed across the different diseases were essential examinations and prescriptions of appropriate drugs. There were some exceptions for patients with specific diseases. For example, accessibility was considered an attractive theme by patients with stroke, dialysis or AMI. In addition, the provision of health education by physicians was necessary for patients with stroke.

**Table 5:** The results of the Kano model for attributes derived from the focus groups for every disease.

	Stroke	Dialysis	AMI	Diabetes
Medical professionalism	O (4/6)	O (2/5)	O (4/6)	O (6/6)
Communication skills	O (4/6)	O (3/5)	O (5/6)	O (4/6)
Facility distance	A (3/6)	A (4/5)	A (4/6)	
Health education (by physician)	M (3/6)			O (5/6)
Health education (by medical staff)				O (5/6)
Execution of the necessary examinations ^a^	M (3/6)	M (4/5)	M (4/6)	M (4/6)
Prescription of appropriate drugs ^a^	M (4/6)	M (4/5)	M (5/6)	M (3/6)

Note: M: must; O: expected; A: attractive; AMI: acute myocardial infarction.

^a^: These ates do not derive from the in-depth interviews, and they were added by the researchers, except for the attribute of facility distance

## 4 Discussion

This is the first study to compare information on public disclosure for different diseases and the first to compile the information patients are likely to value when changing physicians in certain specific contexts using a qualitative approach. In addition, based on the findings above, we prioritized the information according to the Kano model. Previous studies have proposed a framework for the use of report cards and have treated tailored quality information as an important strategy for enhancing the effectiveness of report cards [7, 8]. However, based on our findings, there is somewhat consistent essential quality information across different diseases and in diverse contexts with the exception of specific information for a few diseases. Our study revealed that the top 3 most common information content items for reporting cards were medical professionalism, physician communication skills and accessibility in the general context across most diseases or conditions. Less information was valued by patients with specific diseases, namely, health education by physicians for stroke and for diabetes, health education by medical staff for diabetes, and facility/physician reputation and emotional support for TKR. In addition, we observed that patients focused on specific and mutually relevant information in certain contexts, regardless of the disease except stroke, such as the importance of accessibility, medical professionalism, and physician communication skills/physician attitudes, when patients were in the following specific contexts: changing jobs or relocating, an increase in severity/comorbidity, or conflict with physicians, which are similar to the 3 most common information types mentioned above in the general context. In addition, patients with chronic diseases pay attention to facility fame/reputation and fame while changing the aforementioned context. Finally, regarding the priorities of information in the general context, we also found that clinical process measures and the prescription of appropriate drugs are treated as basic information or necessities; however, most of the aforementioned important information is treated as expected information regardless of the disease.

Our above results of in-depth interviews from qualitative procedures meet the standards for reporting qualitative research [41, 42], and the sensitivity of these themes between the experts and patients ranged from 0.67 to 0.78, which is similar to the findings of another study [30] and demonstrated good categorization. Hence, we believe that the assignment of the statements in the in-depth interviews to specific themes by the experts in this study is valid for every disease or condition.

One study related to the need for information on report cards demonstrated that accessibility, communication with providers and continuity of information were identified as the most needed information [26]; however, this study focused mainly on primary care and did not address the needs of patients with different diseases. In our study, we realized that there was indeed general information that patients valued across diseases in the general context. The top 3 quality pieces of information across most diseases or conditions in the general context in this study are similar to those identified in previous studies as important themes for a single disease or condition generally, namely, medical professionalism [14, 23, 43], communication skills [15, 19, 23, 26, 40, 44–46] and accessibility [25, 26]. In addition, we found that only a few themes varied by disease or condition and need to be tailored in the report card system. Previous studies have demonstrated that physician‒patient communication, bedside support and interaction, and physician reputation are valued by patients undergoing elective surgeries [45, 46]. Our study also revealed similar results: patients undergoing TKR valued emotional support, and facility fame/reputation was more important.

We also found that there is important general information that patients value across diseases in a specific context, and this information is relevant to the context. For example, in the general context, medical professionalism is valued the most regardless of disease, while in the specific context of relocation, most patients value ‘accessibility’ related to the aforementioned context regardless of the disease. These empirical findings could be explained by the fact that the information required by patients is easily influenced by the context [16]. In addition, we found that the reputation of the facility/physician was highlighted, particularly for patients with nonurgent diseases who were willing to change their original physician to an unfamiliar physician. These patients were more likely than patients with catastrophic diseases (e.g., AMI or stroke) to have first searched the facility or physician to determine their reputation because a good reputation may represent good overall quality in hospital and doctor rankings [47, 48]. Finally, we found that patients who have had strokes still value medical professionalism, whether in the context of changing jobs or residences or a conflict with a physician. The reason for this was provided by the patients’ in-depth interviews (in the general context). Patients with stroke are concerned about recovering from their illness or reducing its adverse events and communication regarding drug use, all of which require a competent physician. Hence, regardless of whether a patient is changing residences or has conflicts with physicians, they still consider medical professionalism important. All of these findings are germane to potential theory construction and should be further investigated by quantitative studies.

Regarding the priority of the top themes for every disease, the Kano model revealed that none of them were indifferent themes. In addition, we found that objective quality information, execution of necessary examinations and prescription of appropriate drugs were not significant themes for patients with every disease (no one mentioned these concepts in the in-depth interviews). However, they are basic themes (must haves), as verified by the Kano model, and they expect a physician to have this quality; if a physician does not have it, it is considered unacceptable [49]. Based on this, the technical quality information should be disclosed first, followed by other expected themes, where most of the top themes the patients mentioned in the in-depth interviews were categorized. One theme for the accessibility of critical care (i.e., for patients with AMI and stroke) is even perceived as attractive, which means that it is an optional quality for patients. However, if a nearby hospital has the capacity to perform treatment for recurrent AMI events, patient satisfaction will substantially increase, but if not, patients can tolerate a capable hospital located far from home.

### 4.1 Practical implications

Because the technical process quality, such as prescription and examination, is treated as basic and considerable quality, the report card should disclose interpersonal and technical quality simultaneously. For easy comprehension of technical quality, the burden of reading could be reduced by the use of composite scores, green‒red lights, or star symbols [50, 51]. Regarding interpersonal quality, report cards can provide the most important quality information that all patients value regardless of their disease status, such as medical professionalism, physicians’ communication skills and accessibility. Accessibility can be realized by the software function, which can determine proximity to one’s home, job location, or any other place. Information should be tailored only for specific diseases, such as health education quality for patients with diabetes and stroke. In addition, patients can further search for quality information based on specific contexts, such as the in the context of increasing severity/comorbidity, patients can search for medical professionalism.

### 4.2 Study limitations

There were some limitations in this study. First, the best method for understanding choice behavior in a specific context is observation; however, it is difficult to observe the behaviors studied herein. For the in-depth interviews, we prompted patients to imagine that they were in specific contexts and made sure they wanted to change physicians and asked the same qualitative questions. We believe that their responses reflected their future intentions regarding the choice behavior. Second, regarding the Kano model questionnaire, we did not have many samples to survey, and because the results were from a total of only 22 patients, bias may have occurred. However, to further explore the results from the first stage, i.e., the in-depth interviews, we decided to include the 4 focus groups for specific diseases. We tentatively identified the relative priority of these top themes. All of these findings pertinent to potential theory construction should be further investigated by large-sample quantitative studies relying on the Kano model. Third, the inclusion of primary caregivers on behalf of patients may have affected the results. However, in the focus group, the 5 primary caregivers were there with the elderly patients they cared for. If they did not have the confidence to answer the questions, they were required to ask nearby patients in order to form their answers. Hence, we believe that a certain level of bias could be mitigated. Finally, the exclusion of the TKR procedure from the focus group in the second phase was due to the inability to recruit a sufficient number of participants. One of the objectives of the second-phase focus group was to validate the findings of the first phase’s in-depth interviews. However, since validation of the TKR procedure was not conducted in the second phase, consistency verification of the findings from the first phase’s in-depth interviews regarding TKR was not feasible.

### 4.3 Conclusion

There is common important interpersonal information on report cards that patients may value regardless of their disease. The required information may change depending on the context. According to the results of the Kano model, technical quality should be disclosed first, followed by interpersonal quality information.

## Supporting information

S1 Appendix Kano model. (DOCX)
