# Supplementary material for: Information usefulness of public disclosure in Taiwan: Does it vary across specific diseases/conditions and contexts?
Source: PLoS One. 2025 Mar 28;20(3):e0310340. doi: 10.1371/journal.pone.0310340 (PMC11952214; doi:10.1371/journal.pone.0310340)
Supplement: S1 Appendix — (DOCX) [file pone.0310340.s001.docx]

**Kano model**

Kano model categorizes quality attributes into four types and satisfaction into two types (positive satisfaction and negative dissatisfaction). The first type of quality attribute is Indifferent Quality, which means that regardless of whether high quality is achieved, overall satisfaction does not increase, nor does it cause dissatisfaction. The second type of quality attribute is Must-Be Quality, which means that the quality attribute is an essential element that must be achieved; if achieved, it does not lead to significant satisfaction, but if not achieved, it can result in dissatisfaction. The third type of quality attribute is the one-dimensional quality element, also known as Expected Quality; achieving this quality attribute can lead to satisfaction, while not achieving it can lead to dissatisfaction. The fourth type of quality attribute is Attractive Quality, which, if achieved, generates significant positive satisfaction, but it is acceptable even if not achieved[1]. Kano model provides a priority sequence for executing quality attributes. Generally, Must-Be Quality is the highest priority, followed by Expected Quality, and Attractive Quality is pursued if resources allow.

**References**

1 Kano N, Seraku N, Takahashi F, Tsuji S. Attractive quality and must-be quality. J Jpn Soc Qual Control; 1984; 149(2):39–48.
